# “Covid arm”: Abnormal side effect after Moderna COVID‐19 vaccine

**DOI:** 10.1111/dth.15197

**Published:** 2021-11-16

**Authors:** Vincenzo Picone, Fabrizio Martora, Gabriella Fabbrocini, Laura Marano

**Affiliations:** ^1^ Dermatology Unit, Department of Clinical Medicine and Surgery University of Naples Federico II Naples Italy

Dear Editor

In December 2020 the FDA authorized Pfizer/BioNTech (BNT162b2) and Moderna (mRNA‐1273) COVID‐19 vaccines. From December 2020 through June 2021, according to most recent data, over than 2.4 billion doses of COVID‐19 vaccine have been administrated globally (458 million fully vaccinated, that is 5.9% of global population) over the world. Numerous adverse reactions after messenger RNA (mRNA)‐based COVID‐19 vaccines have been reported. Both of them (Pfizer and Moderna) can be associated with cutaneous adverse effects. Several cutaneous reactions to Moderna (83%) or Pfizer (17%) COVID‐19 vaccines were reported by McMahon et al. for 414 unique patients.^1^ The most common cutaneous reaction was delayed large local reaction, followed by local injection site reactions, urticaria, morbilliform eruptions, erythromelalgia, and pernio/chilblains. We report two cases of “COVID arm”: a localized erythematous reaction at the injection site after the first administration of Moderna COVID‐19 vaccine.^2^ None of them had COVID‐19 infection symptoms prior to their COVID‐19 vaccinations and the onset of their skin rash. In Table S1 are reported patients' clinical data and management.

Patient 1 was a 60‐years‐old man who came at our department presenting a monolateral erythematous rash on his left arm (Figure 1A). The patient reported the appearance of the rash about 30 days earlier, 1 week after administration of the dose one of Moderna COVID 19 vaccine. No associated symptoms and no systemic involvement were reported. On dermatological physical examination it was appreciated a diffuse, pinkish‐colored erythematous patch along the entire surface of the left arm. Diascopy was negative.

**FIGURE 1:**
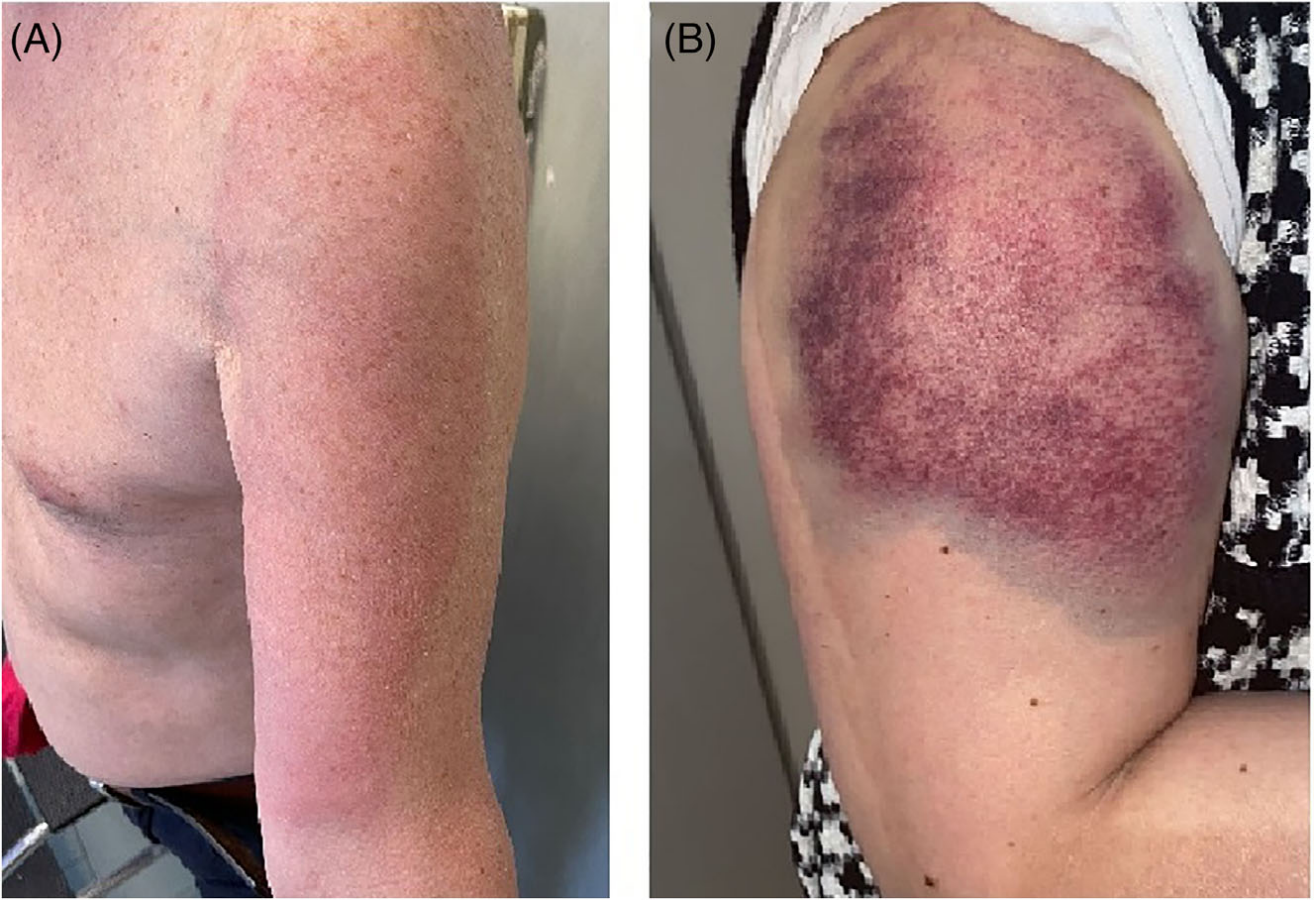
**(**A) Erythematous rash involving the entire surface of the left arm. (B) Erythematous purpuric rash in the right deltoid region

Patient 2 was a 62‐years‐old woman who developed a monolateral giant erythematous rash in the right deltoid region (Figure 1B). During the collection of the anamnesis, it emerged that the patient received the first dose of Moderna COVID 19 vaccine 7 days before the skin eruption. Soreness and burning pain were reported in the site of skin eruption, with no systemic involvement. On dermatological physical examination it was appreciated a purpuric rash with violet edge of dimensions of about 10 cm × 7 cm. It could not have just resulted from bleeding following the vaccination because it appeared 1 week after the dose administration, in addition, the laboratory examination showed elevated inflammatory markers. Diascopy was negative.

ANA, ANCA and complement, serum electrophoresis and blood count were performed in both patients, showing no alterations. For both of them a corticosteroid topic treatment was prescribed with emollients for 10 days. During the follow up visit, after 2 weeks, patient 1 showed an almost complete resolution of the rash, so it was started a gradual suspension of corticosteroid topic treatment. In patient 2 it was observed a partial improvement, with centripetal resolution of the skin eruption so corticosteroid topic therapy was continued for another week. “COVID arm” is an adverse event that may occur at the site of vaccine injection 7–10 days after the first administration of the Moderna COVID 19 vaccine, with the appearance of an erythematous rash associated or not with algic symptoms.^2^ Currently in the literature there are no reported cases of “Covid arm” after the first dose of other vaccines used in the vaccination campaign against SARS‐Cov2. Nevertheless the underlying pathophysiological mechanism remains still unknown.^3^ It is possible that this kind of m‐RNA vaccines cause endothelial damage to microcirculation, responsible for blood extravasation and purpuric lesions.^4^ In several studies it has been reported that an activation of T‐lymphocytes after m‐RNA vaccine is the main cause of endothelial damage.^5^ In conclusion, about our experience, “Covid arm” is a benign adverse event that resolves in a few weeks and does not represent a contraindication to the second dose of the vaccine; however, further studies are necessary to confirm our hypothesis (Informed consent: The patient gave the consent for photo acquisition and publication).

## CONFLICT OF INTEREST

Gabriella Fabbrocini acted as a speaker or consultant for Abbvie, Amgen, Eli Lilly, Janssen, Leo‐Pharma, Almyrall, Novartis, and UCB. None of the contributing authors has any conflict of interest, including specific financial interests of relationships and affiliation relevant to the subject matter or discussed materials in the manuscript.

## Supporting information


**Table S1** Clinical Features And Management.

## Data Availability

The data that support the findings of this study are available on request from the corresponding author. The data are not publicly available due to privacy or ethical restrictions. Data sharing not applicable to this article as no datasets were generated or analyzed during the current study.
