# Supplementary material for: “Covid arm”: Abnormal side effect after Moderna COVID‐19 vaccine
Source: Dermatol Ther. 2021 Nov 16;35(1):e15197. doi: 10.1111/dth.15197 (PMC8646724; doi:10.1111/dth.15197)
Supplement: Supplementary file 1 — Table S1 Clinical Features And Management. [file DTH-35-0-s001.docx]

|  | **Patient 1** | **Patient 2** |
| --- | --- | --- |
| ***Age*** | 60 | 62 |
| ***Sex*** | M | F |
| ***Comorbidities*** | Hypertension, Diabetes,  previous TIA (2018) | Juvenile epilepsy,  Hashimoto thyroiditis |
| ***Vaccine*** | MODERNA | MODERNA |
| ***Dose of Vaccine*** | First | First |
| ***Time to onset of rash*** | 1 week | 1 week |
| ***Diascopy*** | Negative | Negative |
| ***COVID19 PCR*** | Negative | Negative |
| ***ANA ANCA Complement*** | Negative/normal | Negative/normal |
| ***Therapy*** | Topical corticosteroids | Topical corticosteroids |
| ***Time of resolution*** | 2 weeks | 3 weeks |

**Supplementary Table 1 : CLINICAL FEATURES AND MANAGEMENT**
